# Bacillus paralicheniformis isolated from a hypersaline sediment of Lake Texcoco, draft genome sequence of strain TXO7B-1SG6

**DOI:** 10.1128/mra.00338-22

**Published:** 2022-07-27

**Authors:** Lorna Catalina Can-Ubando, Ayixon Sánchez-Reyes, Gauddy Lizeth Manzanares-Leal, Keila Isaac-Olivé, Horacio Sandoval-Trujillo, Ninfa Ramírez-Durán

**Affiliations:** a Laboratorio de Investigación en Microbiología Médica y Ambiental, Facultad de Medicina, Universidad Autónoma del Estado de México, Toluca, México, México; b Investigador por México, CONACYT—Instituto de Biotecnología, Universidad Nacional Autónoma de México, Cuernavaca, Morelos, México; c Laboratorio de Investigación en Teranóstica, Facultad de Medicina, Universidad Autónoma del Estado de México, Toluca, México, México; d Laboratorio de Producción de Biológicos, Departamento de Sistemas Biológicos, Universidad Autónoma Metropolitana—Xochimilco, Ciudad de México, México; University of Rochester School of Medicine and Dentistry

## Abstract

We present the draft genome sequence of the halotolerant strain Bacillus paralicheniformis TXO7B-1SG6.

## ANNOUNCEMENT

Bacillus paralicheniformis, a Gram-positive bacillus cultured in medium with up to 10% NaCl, was described as a new species in 2015 (1). It controls plant pathogens and produces bioactive secondary metabolites (2). We present the draft genome sequence of strain TXO7B-1SG6, isolated from hypersaline sediment from Lake Texcoco, Mexico (19°28′42″N, 98°58′26'″W), in June 2019 and identified as Bacillus paralicheniformis.

Sediment solutions (0.1 mg/mL concentration) were prepared for isolation. The dilutions were inoculated onto petri dishes using Czapek culture medium and brain heart infusion (BHI) agar (37°C, 7 to 10 days). The strain was isolated and cultured on halophilic medium (HM) (3), being halotolerant (NaCl growth range, 0 to 5%; optimum, 5%) with a pH range of 7 to 10 (optimum 8).

DNA was extracted using the Promega Wizard genomic kit (A1125). The 16S rRNA gene was amplified by PCR with the universal primers 27F and 1492R (4) and sequenced by Psomagen (MD, USA). A consensus sequence (1,436 bp) was obtained using the BioEdit v7.09 program. BLAST (5) and the EzBioCloud database (6) were used for sequence comparison. The highest identity percentages were found with Bacillus haynesii (97.63%; BLAST) and Bacillus paralicheniformis (98.52%; EzBioCloud).

To sequence the TXO7B-1SG6 genome, the strain was inoculated onto HM agar to obtain biomass. High-molecular-weight (HMW) DNA was extracted using the Quick-DNA HMW MagBead kit (Zymo Research catalog number D6060). The sample was subjected to long-read sequencing using the MinION platform (ONT) of the Institute of Biotechnology at the National Autonomous University of Mexico (IBT-UNAM).

A MinION library was prepared from unsheared genomic DNA using the ligation sequencing kit (SQK-LSK109). The EXP-NBD104 barcoding kit was used for multiplexing and the R9.4.1 flow cell for sequencing. The reads were base called and demultiplexed using Guppy v4.4.1 (7). Raw read quality exploration using NanoPlot showed 138,694 reads with a mean length of 1,608.8 nucleotides (nt) and an *N*_50_ value of 12,283 nt. The reads were quality filtered using Filtlong v0.2.1 (8), and adapters were trimmed using Porechop v0.2.4 (9). A total of 138,694 reads were obtained with a mean read quality of 12.5. The filtered reads were assembled using Unicycler v0.4.8 software (option *–l long-read-only assembly*) (10), and the final genome was rotated (the *dnaA* sequence was set to the first nucleotide position) using the Circlator v1.5.5 pipeline (11). The genome was annotated using the NCBI Prokaryotic Genome Annotation Pipeline (PGAP) v6.1 (12). Default parameters were used for all software unless otherwise noted.

A chromosome-level assembly was achieved, resulting in 1 single replicon with a size of 4,523,282 bp, a 45.60% G+C content, and an average read sequencing coverage depth of 50×. The completeness and contamination were estimated using CheckM v1.1.1 (13) at 94.5% and 0.3%, respectively. The average nucleotide identity (ANI) was estimated according to the NCBI workflow (14). The genome of TXO7B-1SG6 has an ANI of 98.72% to *B. paralicheniformis* strain KJ-16 (GenBank accession number GCA_001042485.2).

A total of 4,159 coding genes were identified, including 4 noncoding RNAs (ncRNAs), 24 rRNAs (8 5S, 8 16S, and 8 23S), and 82 tRNAs.

### Data availability.

The consensus sequence of the 16S rRNA gene has been deposited at GenBank under accession number OM746865. The draft genome sequence has been deposited at NCBI GenBank under accession number CP082897; the version described in this article is version CP082897.3. The associated BioSample accession number is SAMN20057199, and the BioProject accession number is PRJNA743662. The raw reads were deposited at the SRA under accession number SRR18345719.
